# Administrative prevalence and incidence, characteristics and prescription patterns of patients with migraine in Germany: a retrospective claims data analysis

**DOI:** 10.1186/s10194-020-01154-x

**Published:** 2020-07-06

**Authors:** Tatjana Roessler, Juergen Zschocke, Anne Roehrig, Michael Friedrichs, Heiko Friedel, Zaza Katsarava

**Affiliations:** 1 Lilly Deutschland GmbH, Werner-Reimers-Straße 2-4, 61352 Bad Homburg vor der Höhe, Germany; 2 Team Gesundheit, Gutleutstr. 163-167, 60327 Frankfurt am Main, Germany; 3 Team Gesundheit, Rellinghauser Str. 93, 45128 Essen, Germany; 4 Evangelical Hospital Unna, Unna, Germany; 5 Department of Neurology, University of Duisburg-Essen, Essen, Germany; 6 EVEX Medical Corporation, Tbilisi, Republic of Georgia; 7 IM Sechenov First Moscow State Medical University (Sechenov University), Moscow, Russian Federation

**Keywords:** Migraine, Germany, Administrative prevalence, Administrative incidence, Comorbidity, Prescription patterns

## Abstract

**Background:**

Migraine is a frequent headache disorder with high disease burden. The aims of this study were to determine the administrative prevalence and incidence of migraine in Germany; and to elucidate disease characteristics, prescription patterns and the patient journey through the German healthcare system.

**Methods:**

In this retrospective, observational study, adult patients with migraine (International Classification of Diseases, 10th revision, German modification G43) were identified in the anonymised German Company Sickness Fund database (CSFD) from 2008 through 2016. The administrative prevalence and incidence of migraine were calculated for the total CSFD study population and extrapolated to the German Statutory Health Insurance (SHI) population. Migraine subtypes, concurrent diagnoses, prescription patterns and visited healthcare professional groups were analysed.

**Results:**

A total of 243,471 patients with migraine were identified in the CSFD (2008–2016); 78.0% were female and 45.3% were aged 35–54 years. The administrative prevalence of migraine, extrapolated to the SHI population, ranged between 2.89% in 2008 and 3.98% in 2016; administrative incidence ranged from 0.587% in 2009 to 0.267% in 2016, and varied between 0.399% and 0.442% during 2011 to 2015. Overall, 29.1% of patients received at least one prescription for any preventive medication listed in the German guideline. Only 7.9% received the same preventive medication for more than 1 year, with 82.9% of these patients discontinuing the medication before study end. Regarding acute medications, 74.2% of prescriptions were for analgesics/non-steroidal anti-inflammatory drugs and 21.2% were for triptans. General practitioners most commonly diagnosed and treated migraine in the CSFD population. Patients with prescriptions for two or more different preventive therapy classes had higher use of acute and emergency medications, and visited healthcare professionals and hospitals more frequently than patients with no prescriptions or prescriptions for only one preventive therapy class.

**Conclusions:**

The administrative prevalence of migraine in this claims database suggests many patients with migraine did not seek medical care. Of those who did, fewer than one-third received preventive medication, with most patients having been prescribed only one such medication and few having continued treatment beyond 1 year. These outcomes suggest there is scope for improvement in migraine management in Germany.

## Background

Migraine is a disabling primary headache disorder [1] characterised by headaches and associated symptoms including aura, photophobia, phonophobia and nausea/vomiting [2, 3], which can lead to considerable disruption of the professional and private lives of affected individuals [4, 5].

The diagnosis of migraine is based on clinical history and a structured exclusion of secondary headache, using the International Classification of Headache Disorders (ICHD) criteria [1]. Migraine is also accompanied by a number of comorbidities, such as psychiatric disorders (e.g. depression and anxiety), allergy, asthma, sleep disorders, fatigue and cardiovascular diseases [3, 4, 6].

The treatment of patients with migraine aims to relieve pain or limit the attack, reduce disability and restore function, improve health-related quality of life and manage comorbidities [2, 7]. Pharmacological therapy comprises both acute and preventive treatments [8]. Acute treatment options include analgesics (foremost non-steroidal anti-inflammatory drugs [NSAIDs]), antiemetics (e.g. metoclopramide), triptans (e.g. almotriptan, oral or subcutaneous sumatriptan) and emergency medications (e.g. intravenous metoclopramide, subcutaneous sumatriptan) [8]. Triptans, as a class of specific migraine medications, are the first choice for patients who have had an inadequate response to non-migraine specific analgesics [8]. Opioids and ergotamines take a special position in the acute treatment algorithm, and they are generally not recommended by the guideline of the German Migraine and Headache Society and the German Society of Neurology (hereafter referred to as the German guideline for migraine) for reasons of toxicity and other adverse effects [8].

**Fig. 1:**
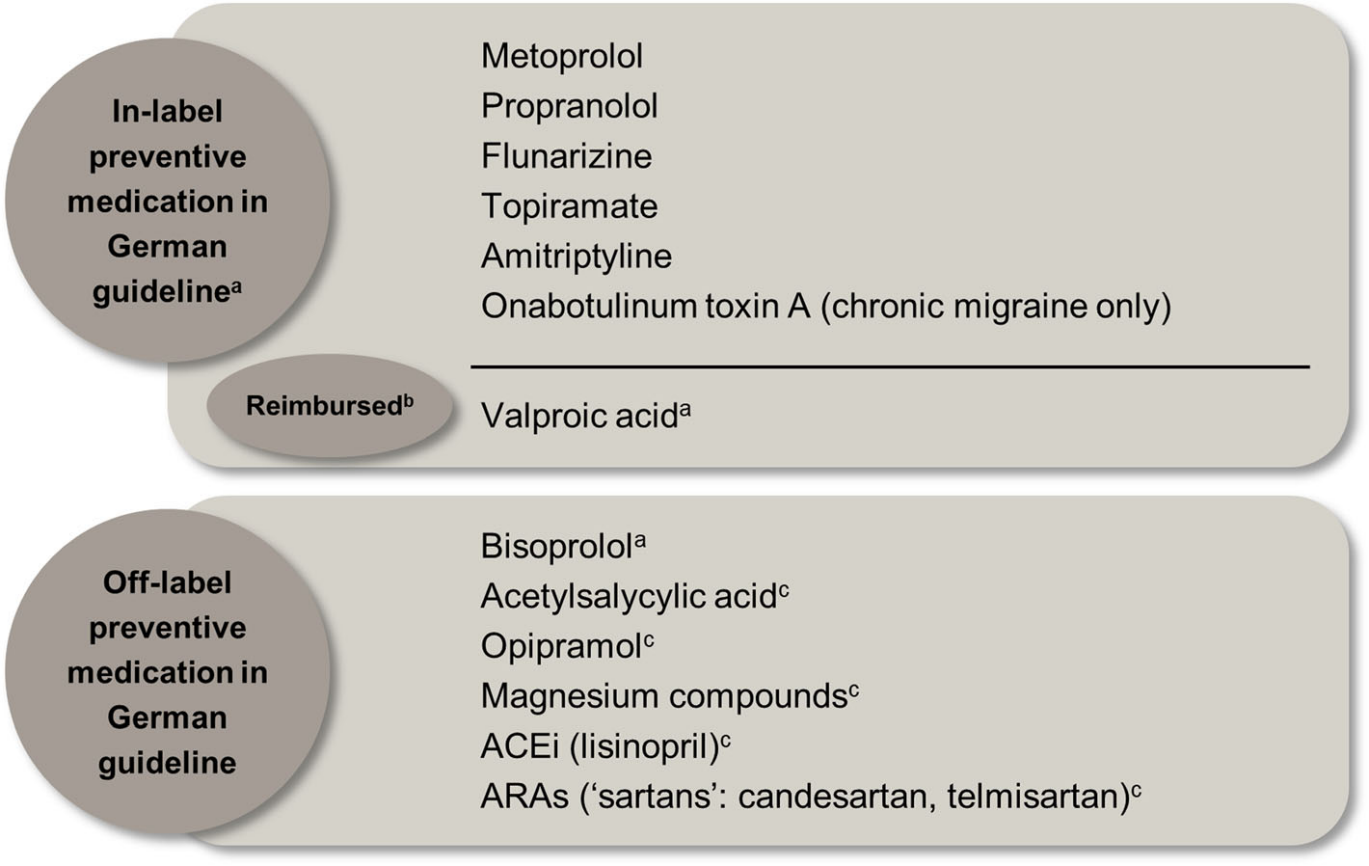
Preventive medication according to the German guideline for migraine [8]. ^a^Preventive medication(s) with good evidence. ^b^Off-label, but may be prescribed at the expense of the German Health Care System (Social Health Insurance) pursuant to Annex VI, Section K of the German medicinal product directive. ^c^Preventive medication with lower evidence. In the current analyses, Group 1 preventive medications are in-label preventive medications plus valproic acid; Group 2 preventive medications are all preventive treatments according to the German guideline for migraine (in- and off-label). *ACEi* angiotensin-converting enzyme inhibitor; *ARA* angiotensin II receptor antagonist

Preventive medications are primarily used to reduce the attack frequency [8]. According to the German guideline, the decision to administer preventive medications should be driven by the frequency of monthly migraine attacks, significant impairment in health-related quality of life and a high risk of medication overuse [8]. In Germany, approved conventional preventive medications (medication classes) are propranolol and metoprolol (beta-blockers), flunarizine (calcium channel blocker), topiramate (anticonvulsant), amitriptyline (antidepressant) and onabotulinum toxin A (muscle relaxant; in chronic migraine only) [8]. Most recently – and outside the scope of these analyses – erenumab, galcanezumab and fremanezumab (calcitonin gene-related peptide [CGRP] inhibitors) were approved in 2018 or 2019 [9–11]. Valproic acid, although off-label, may be prescribed at the expense of the German Statutory Health Insurance (SHI) pursuant to Annex VI, Section K of the German Medicinal Product Directive. In addition to the approved preventive medications mentioned here, a variety of other medicines are listed in the German guideline for migraine, although they are not approved for migraine in Germany. These include bisoprolol (good evidence for use according to the German guideline for migraine) and agents with less scientific evidence to support their use (opipramol, acetylsalicylic acid, magnesium, magnesium plus vitamin B2 plus coenzyme Q10, angiotensin-converting enzyme inhibitors [ACEis], specifically lisinopril, and the angiotensin II receptor antagonists [ARAs; ‘sartans’] candesartan and telmisartan) (Fig. 1) [8].

Prevalence data for migraine have been obtained using population-based studies [12, 13]. Migraine was reported to have a crude global prevalence of 11.6% (11.4% in Europe) and occurred more frequently in women than men (globally: 13.8% vs 6.9%) [14]. In Germany, the prevalence of migraine was estimated to be 10.6% (women: 15.6% vs men: 5.3%), according to a telephone survey (*n* = 7341) conducted in 2004 [12], and 13.4% (19.1% vs 7.1%), according to a population-based, longitudinal cohort survey (*n* = 9944) conducted from 2003 to 2005 [13].

It has been reported that the majority of patients with migraine do not consult a healthcare professional [15]. It is therefore of interest to ascertain how many patients consult healthcare professionals because of their migraine and to further characterise these patients with regard to the treatments received and their healthcare system interaction. Analyses based on claims data are important and powerful sources of information to assist decision making for healthcare stakeholders, researchers and policy makers, and are supported by the German government [16].

The aims of this study – based on administrative claims data – were therefore multifaceted: to describe the administrative prevalence and incidence of migraine in Germany; to provide information on the characteristics of and prescription patterns in patients with migraine; and to determine which healthcare professionals were involved in the diagnosis and continued treatment of these patients. Additionally, the study aimed to describe whether patients prescribed more preventive treatments differ in utilisation patterns and healthcare professional contacts from those prescribed fewer or no preventive treatments.

## Methods

### Study design and data source

This was a retrospective, observational study of anonymised data from a database of various nationwide German Company Sickness Funds (Betriebskrankenkassen; CSFD) over the 9-year period from 2008 to 2016. This CSFD merges data from a number of large company sickness funds that are providers within the German SHI system (the Gesetzliche Krankenversicherung). The sample contained approximately 5.6 million insured persons over the 9-year study period, with approximately 4.3 million people included in 2016. The CSFD sample is representative of the German SHI and has been shown to include a population with a similar age and gender structure to that of the 71.4 million people covered by the SHI (Supplementary Figure 1).

The CSFD contains longitudinal information of insured persons with respect to all areas of services refunded by the SHI. It includes detailed data on: in- and outpatient care; sick-leave and benefits based on the International Classification of Diseases, 10th revision; German modification (ICD-10-GM) diagnoses [17]; drug prescriptions (Anatomical Therapeutic Chemical [ATC] code of prescribed medications and related costs, *Pharmazentralnummer* [PZN; German drug identifier]); and the date of prescription, medical aids and registration data (age, gender, time insured, region, insurance status and level of education of those insured). As is usual for these types of data, information on drug prescriptions and hospitalisations was documented on a daily basis, and diagnoses made by the healthcare professional group were available on a quarterly basis. The database allows the analysis of groups of patients with defined characteristics (e.g. those with a specific disease or prescriptions of specific medications, or combinations of characteristics) at a specific date (index) and comparisons between groups.

Access to these strictly regulated data was requested and obtained from the CSFD, who had no other involvement in the analyses. Data from the electronic databases of the collaborating anonymised SHI funds were gathered under naturalistic conditions and anonymised by the providers in accordance with an approved data privacy concept. The raw data were imported, prepared and checked by the authors using previously established processes. Use of the anonymised study database for health services research was fully compliant with German federal law; therefore, International Review Board/ethical approval was not needed.

### Identification of the target population

Patients meeting the following inclusion criteria during the study period from 1 January 2008 to 31 December 2016 were identified. Outpatients were required to have an assured migraine diagnosis (ICD-10-GM code G43, supplemented by ‘G’ [‘gesichert’ or assured] and ‘Z’ [‘Zustand nach’ or condition after; i.e. the patient had this diagnosis earlier and it continues to affect their health]). Inpatients or patients identified from sick leave data were included based on the principal migraine diagnosis (ICD-10-GM 43.-) made by the treating physician.

The first recorded migraine diagnosis defined the index year/quarter; however, an outpatient migraine diagnosis was considered confirmed only if there was a following migraine diagnosis within 1 year but in a different quarter (M2Q criterion: ‘Mindestens zwei [2] Quartale’, which translates to ‘at least two [2] quarters’).

Eligible patients were adults (aged at least 18 years) who had an interval of continuous enrolment in the CSFD during the study period. Follow-up (time period subsequent to the index date) was of variable length, with each person followed-up until (i) discontinuation of continuous enrolment, (ii) death or (iii) the end of the study period (31 December 2016) – whichever was earliest.

### Data collection

Demographic characteristics (age and gender) were retrieved in the index year. Age was categorised in seven groups (18–24, 25–34, 35–44, 45–54, 55–64, 65–74 and ≥ 75 years). Migraine diagnoses (ICD-10-GM code) and concurrent diagnoses, as well as data regarding the specialty of the healthcare professional who prescribed any treatment, migraine treatments (type and number) and hospitalisations were collected throughout the study period. Medications (Supplementary Table 1; Fig. 1) were considered preventive against migraine only if a diagnosis of migraine was identified within the same quarter in the in- or outpatient, or sick-leave data, as most are approved for several indications.

Preventive medications were categorised into two groups depending on their approval and funding status from 2008 to 2016 in Germany: Group 1, in-label preventive medications plus valproic acid (propranolol, metoprolol, flunarizine, topiramate, amitriptyline, onabotulinum toxin A and valproic acid); Group 2, all preventive medications according to the German guideline for migraine [8] (preventive medications from Group 1 plus bisoprolol, opipramol, lisinopril, ARA therapy and magnesium compounds). The number and duration of phases (periods of time) of continuous treatment with any preventive medication (Group 2) were calculated. Treatment was considered continuous if a follow-up prescription was identified within 60 days of the estimated date of the next prescription of the same medication. The estimated date of the next prescription was calculated by adding the number of days’ supply (amount per prescription divided by the recommended daily dose) to the date of the actual prescription. The number of acute and emergency medications prescribed to patients with migraine in 2016 was also calculated (Supplementary Table 1 lists the acute, emergency and preventive medications considered; based on the German guideline for migraine [8]).

The patient journey was determined by counting the number of visits to healthcare professional groups, the number of hospitalisations and the number of hospital outpatient visits for the index quarter and every following year. These numbers were then divided by the number of patients with a diagnosis in the index quarter/follow-up year, for each year of follow-up, to determine the proportions of patients experiencing each event. Healthcare professional groups included anaesthetists, general practitioners, gynaecologists, internists, nephrologists, neurologists (including neurologist-psychiatrists), ophthalmologists, orthopaedists, otorhinolaryngologists, psychiatrists/psychotherapists and hospitals.

### Data analysis

For each year from 2008 to 2016, the number and percentage of prevalent migraine cases were calculated for the CSFD study population by gender and age. Incident migraine cases in the CSFD were calculated similarly, with an exception for the year 2008: a case was considered incident if the first migraine diagnosis appeared at least 1 year after enrolment. Study sample population (CSFD) estimates of prevalence and incidence underwent age- and gender-adjusted extrapolation to the entire population of the SHI [18]. Administrative prevalence and incidence values are reported as weighted means with corresponding 95% confidence intervals (CIs), based on 54% (2008–2013) or 53% (2014–2016) of the SHI population being female.

Proportions of patients with migraine sub-diagnoses, any concurrent diagnoses, concurrent diagnoses of special interest and preventive medication prescriptions (Group 1 and Group 2) were calculated for the study period (2008–2016). Concurrent diagnoses of special interest were those that were contraindications or involved special warnings and precautions for use of approved preventive medications (Group 1), according to their respective German summary of product characteristics and as listed by Diener et al. [8]. The numbers of acute, emergency and preventive medications prescribed per patient, and the most commonly prescribed preventive medications were identified only for 2016 to ensure that the collected data were as up-to-date as possible. The most frequently visited healthcare professional groups, including the most frequently first-visited healthcare professional groups (as noted earlier, hospitals were included as a specialist group); the number of visits per person to healthcare professionals; and the number of hospitalisations per person each year from 2008 to 2016 were determined. Outcomes were reported for all patients with migraine and for patients grouped according to restricted Group 1 (propranolol, metoprolol, flunarizine, topiramate and amitriptyline, but not onabotulinum toxin A or valproic acid) preventive medication usage (none, only one, at least two such medications from different treatment classes). The healthcare professional first diagnosing the migraine was defined as the professional who first documented a diagnosis of migraine within the index quarter. For each year of follow-up, the number of hospitalisations was divided by the time period that patients were included in the database in the respective year (some patients may have been included for fewer than 12 months) and data were reported as the number of hospitalisations per 100 person-years.

All analyses were descriptive and were performed with appropriate statistical methods using SAS 9.4. Categorical variables were presented as number and percentage of patients; continuous variables were summarised as mean and standard deviation (SD).

## Results

### Sample population

**Fig. 2:**
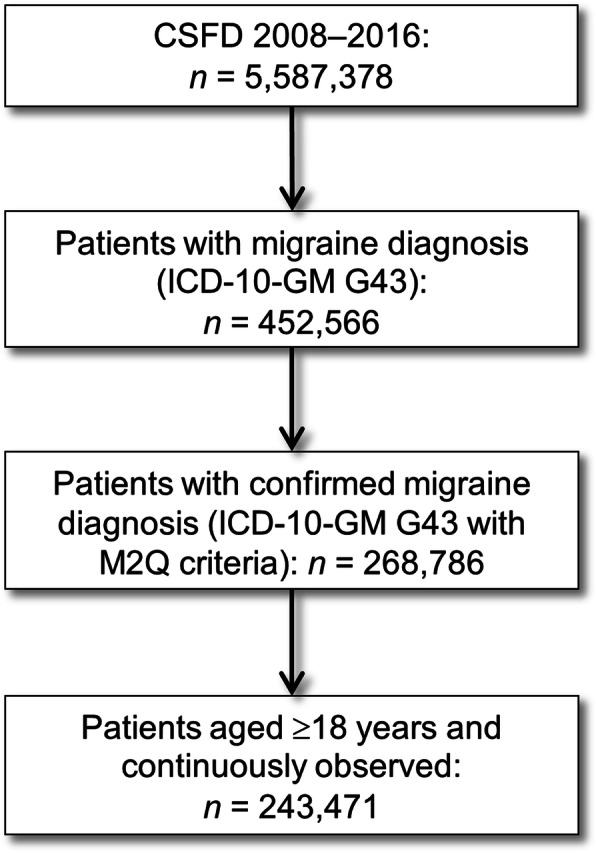
Flow chart of the identification of eligible patients with migraine in the German Company Sickness Fund database (CSFD) 2008 to 2016. *ICD-10-GM* International Classification of Diseases, 10th revision, German modification; M2Q ‘Mindestens zwei [2] Quartale’, which translates to ‘at least two [2] quarters’

**Fig. 3:**
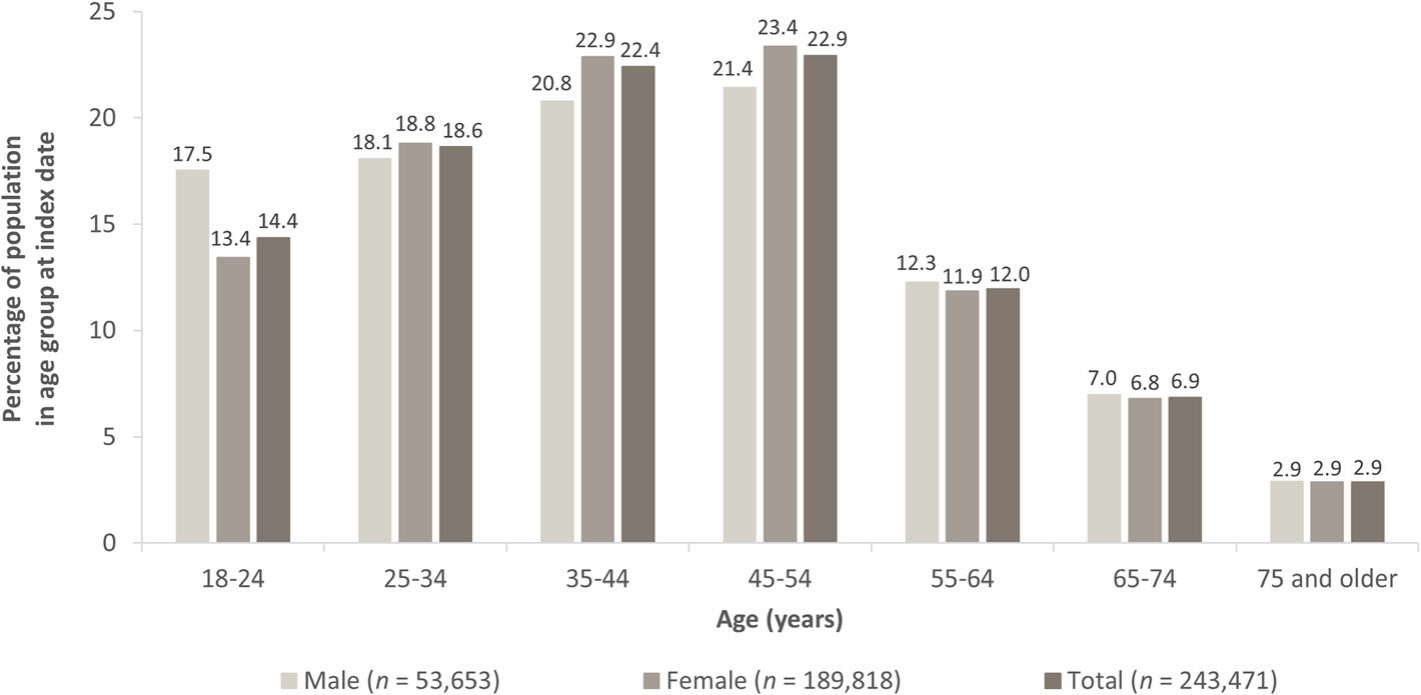
Age distribution of patients with migraine in the German Company Sickness Fund Database 2008 to 2016

The CSFD 2008 to 2016 included a total of 5,587,378 people, with 268,786 patients having a confirmed diagnosis of migraine. Of these, 243,471 patients were aged ≥18 years and continuously observed, and were therefore eligible for analyses (Fig. 2). Most patients were female (*n* = 189,818; 78.0%), resulting in a male to female ratio close to 1:4. The mean (SD) age of these patients was 42.7 (15.4) years, and 45.3% were aged 35 to 54 years (Fig. 3).

Throughout the analysis period, the same ICD-10-GM G43 diagnosis was documented for 63.1% of patients, while at least two different ICD-10-GM G43 diagnoses were documented for 36.9% of patients. A diagnosis of unspecified migraine was documented during the analysis period for the majority of patients (82.4%), followed by migraine with aura, migraine without aura, other migraine and complicated migraine (which included chronic migraine; Table 1). Overall, a diagnosis of complicated migraine (ICD-10-GM G43.3) was documented among other migraine diagnoses for 2.9% of all patients, while solely this diagnosis was documented for 0.4% of all patients.

**Table 1:** Migraine diagnosis by subtype (ICD-10-GM code) in the German Company Sickness Fund Database 2008 to 2016

ICD-10-GM code	Migraine subtype	G43 diagnosis during analysis period (% patients)
G43.0	Migraine without aura (common migraine)	25.5
G43.1	Migraine with aura (classical migraine)	26.1
G43.2	Status migrainosus	1.4
G43.3	Complicated migraine (including chronic migraine)	2.9
G43.8	Other migraine	11.2
G43.9	Migraine, unspecified	82.4

A migraine diagnosis was considered confirmed only if there was a following migraine diagnosis within 1 year but in a different quarter (M2Q criterion: ‘Mindestens zwei [2] Quartale’, which translates to ‘at least two [2] quarters’). Patients could receive more than one kind of G43 diagnosis

*ICD-10-GM* International Classification of Diseases, 10th revision, German modification

### Prevalence and incidence

**Fig. 4:**
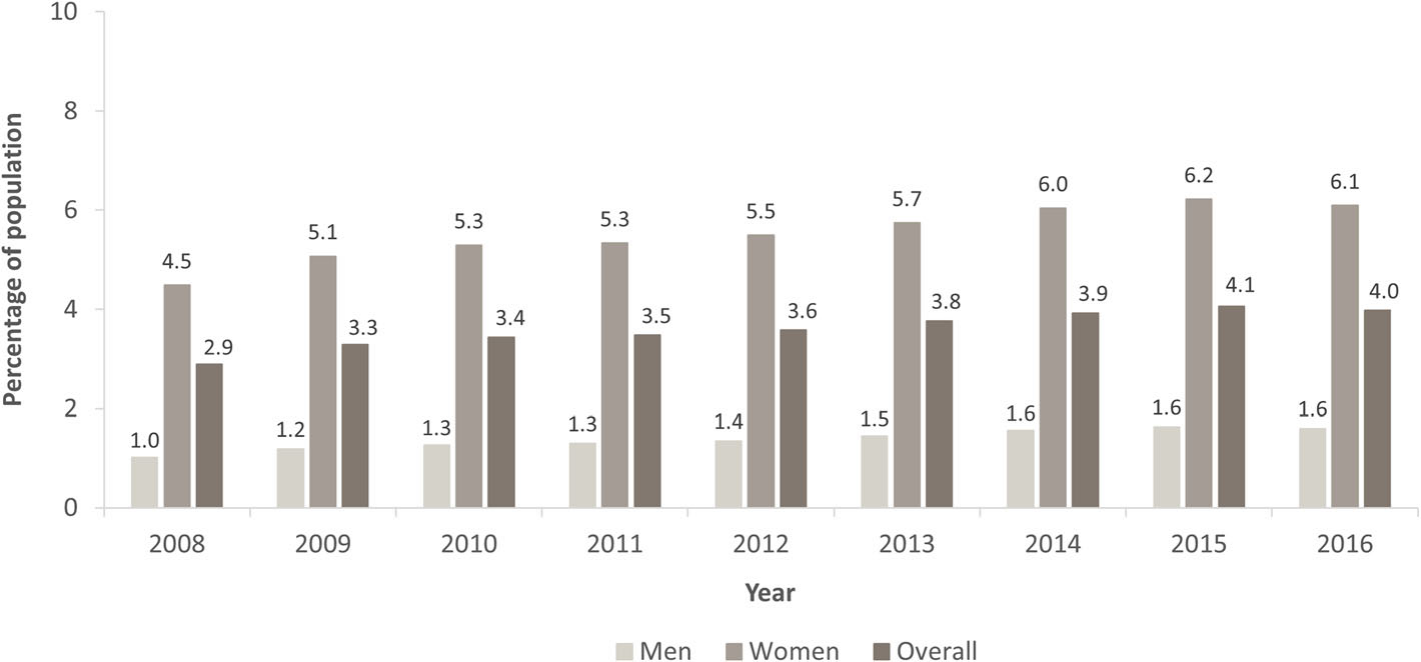
Extrapolated administrative prevalence of migraine over time by sex in the total German Statutory Health Insurance system population. Overall prevalence values are weighted means, adjusted for sex; 95% confidence intervals are not displayed due to their narrow width

The administrative prevalence of migraine in 2016, extrapolated to the total SHI population, was 3.98% (95% CI: 3.97, 3.98), being 1.60% (95% CI: 1.60, 1.61) in men and 6.09% (95% CI: 6.08, 6.09) in women. There was a trend for the administrative prevalence to increase from 2.89% (95% CI: 2.89, 2.90) in 2008 to 4.06% (95% CI: 4.06, 4.07) in 2015 (in men: 1.02% [95% CI: 1.01, 1.02] to 1.63% [95% CI: 1.63, 1.64]; in women: 4.49% [95% CI: 4.49, 4.50] to 6.22% [95% CI: 6.21, 6.23]) (Fig. 4).

**Fig. 5:**
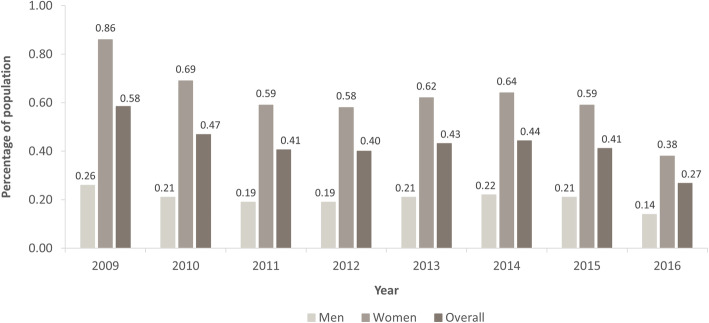
Extrapolated administrative incidence of migraine over time by sex in the total German Statutory Health Insurance system population. Incidence data could not be calculated for 2008 because the first migraine diagnosis needed to appear at least 1 year after enrolment to be considered incident. Overall incidence values are weighted means, adjusted for sex; 95% confidence intervals are not displayed due to their narrow width

The administrative incidence of migraine extrapolated to the SHI population varied between 0.399% (95% CI: 0.397, 0.402) and 0.442% (95% CI: 0.440, 0.445) during the period 2011 to 2015, but tended to decrease from 0.587% (95% CI: 0.584, 0.589) in 2009 to 0.267% (95% CI: 0.265, 0.269) in 2016. Administrative incidence ranged from 0.261% (95% CI: 0.259, 0.263) to 0.136% (95% CI: 0.135, 0.138) in men and from 0.864% (95% CI: 0.861, 0.867) to 0.383% (95% CI: 0.381, 0.385) in women from 2009 to 2016. There was little variation between 2011 and 2015 for either men or women (Fig. 5).

### Concurrent diagnoses

The most common concurrent diagnosis in patients with migraine between 2008 and 2016 was dorsalgia (back pain), followed by acute upper respiratory tract infections, and disorders of refraction and accommodation (Table 2). The most common concurrent diagnoses of special interest, which occurred in > 10% of patients with migraine, are summarised in Table 2, with depressive episodes or recurrent depressive disorders (ICD-10-GM code F32–F33) being the most frequent.

**Table 2:** Concurrent diagnoses in patients with migraine in the German Company Sickness Fund Database 2008 to 2016

ICD-10-GM code	Diagnosis	Men (***n*** = 53,653) (%)	Women (***n*** = 189,818) (%)	Overall (***n*** = 243,471) (%)
**Most common concurrent diagnoses**				
M54	Dorsalgia	78.2	82.7	81.7
J06	Acute upper respiratory infections of multiple and unspecified sites	67.0	66.1	66.3
H52	Disorders of refraction and accommodation	55.6	63.8	61.9
R10	Abdominal and pelvic pain	40.0	64.4	59.0
F45	Somatoform disorders	38.1	55.4	51.6
M99	Biomechanical lesions, not elsewhere classified	45.2	52.0	50.5
I10	Essential (primary) hypertension	43.1	38.5	39.6
N89	Other non-inflammatory disorders of vagina	–	65.6	–^a^
**Concurrent diagnoses of special interest occurring in > 10% of patients in men, women or overall**				
F32–F33	Depressive episode – recurrent depressive disorder^b^	33.4	45.5	42.9
F40–F41	Phobic and other anxiety disorders^c^	17.1	27.5	25.2
J45	Asthma^d^	14.7	17.0	16.5
K70–K77	Diseases of liver^e^	15.7	12.2	13.0
I95	Hypotension^d,f^	6.9	14.3	12.7
I49	Other cardiac arrhythmias^d^	10.8	11.8	11.6
E10–E14	Diabetes mellitus^d^	12.4	10.0	10.6
N40	Hyperplasia of prostate^g^	19.7	–	–^a^

^a^Because this diagnosis could only affect one gender, to provide an overall percentage would be misleading

^b^Contraindication or special warning and precaution for use for beta-blockers, flunarizine and topiramate

^c^Contraindication or special warning and precaution for use for topiramate

^d^Contraindication or special warning and precaution for use for beta-blockers

^e^Contraindication or special warning and precaution for use for valproic acid

^f^Surrogate marker for orthostatic dysregulation

^g^Contraindication or special warning and precaution for use for amitriptyline

Selections of contraindications were based on the guideline by the German Migraine and Headache Society and the German Society of Neurology [8]

*ICD-10-GM* International Classification of Diseases, 10th revision, German modification; − not applicable

### Medication use

**Fig. 6:**
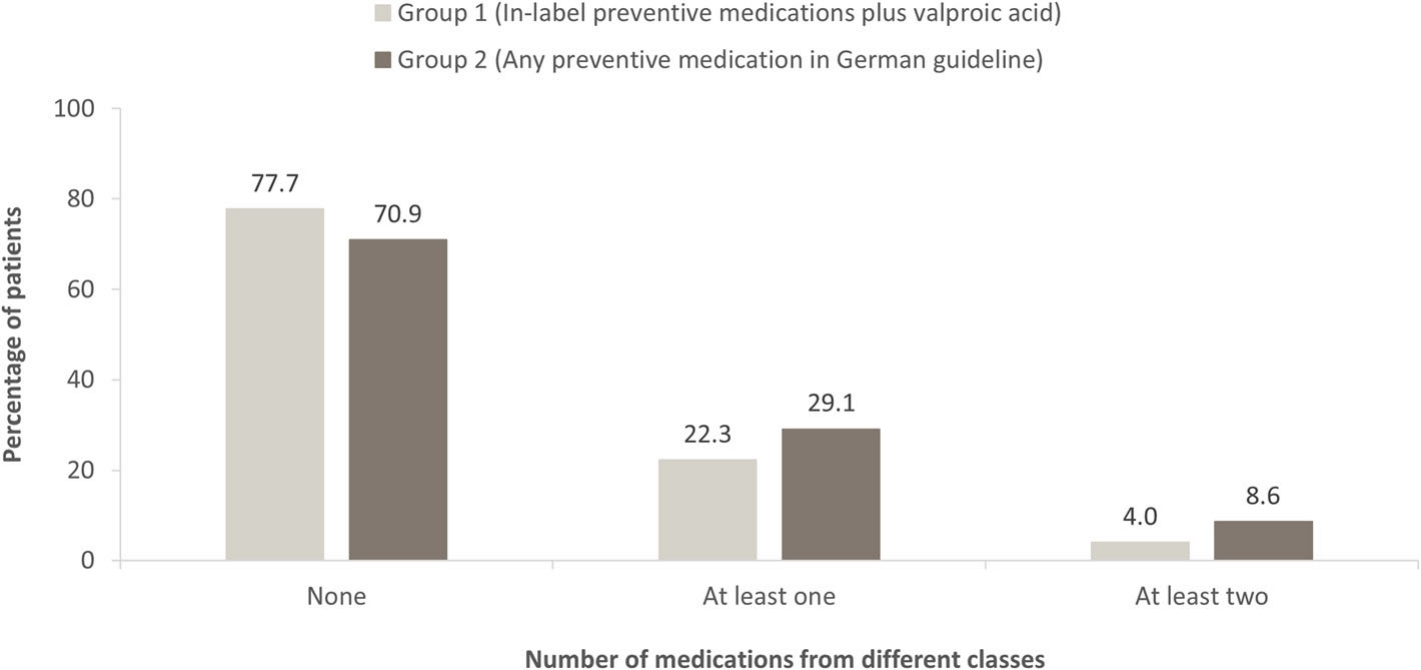
Percentage of patients with no, at least one, or at least two prescriptions of preventive medications from different classes in the German Company Sickness Fund Database 2008 to 2016. Preventive medications considered were Group 1 (in-label preventive medications plus valproic acid, i.e. [metoprolol or propranolol], amitriptyline, flunarizine, topiramate, onabotulinum toxin A or valproic acid) and Group 2 (preventive medications according to German guideline for migraine [10]: Group 1 preventive medications plus opipramol, lisinopril or angiotensin II receptor antagonist [‘sartan’] therapy); magnesium was excluded from these analyses because no defined daily dose was available

Overall, 22.3% of patients with migraine received at least one prescription for in-label preventive medications plus valproic acid (Group 1 preventive medication), and 29.1% received at least one prescription for any preventive medication (i.e. both in- and off-label preventive medications mentioned in the German guideline for migraine; Group 2 preventive medication) (Fig. 6). However, only a small proportion of patients (4.0% of the total sample) had been prescribed two or more in-label preventive medications plus valproic acid (Group 1) from different classes from 2008 to 2016 (Fig. 6); the proportion of patients prescribed two or more Group 2 medications from different classes during this period was somewhat larger (8.6%). The most commonly prescribed Group 2 preventive medications in 2016 were beta-blockers (specifically, metoprolol and bisoprolol) (Table 3). When considering only patients who had been diagnosed with complicated migraine (G43.3, including chronic migraine) at least once during the study period, 38.0% received at least one prescription for in-label preventive medications plus valproic acid (Group 1 preventive medication).

**Table 3:** Prescribed in- and off-label (Group 2) preventive medications for patients with migraine in the German Company Sickness Fund Database 2016

Class Preventive medication	Percentage of all prescriptions for preventive medications
**Beta-blockers**	**53.8**
Metoprolol	29.6
Bisoprolol	21.0
Propranolol	3.2
**ACEis and ARAs**	**22.4**
ARAs (‘sartans’)	20.1
Lisinopril	2.3
**Antidepressants**	**16.0**
Amitriptyline	9.5
Opipramol	6.5
**Anticonvulsants**	**5.7**
Topiramate	4.2
Valproic acid	1.5
**Muscle relaxants**	**0.8**
Onabotulinum toxin A^a^	0.8
**Calcium channel blockers**	**0.8**
Flunarizine	0.8
**Magnesium compounds**	**0.5**

Data shown are for prescribed Group 2 preventive medications (all preventive medications according to the German guideline for migraine [8])

^a^Prescriptions for the Allergan brand of onabotulinum toxin A only were included

*ACEi* angiotensin-converting enzyme inhibitor; *ARA* angiotensin II receptor antagonist

Across the 9-year study period, a mean of 0.13 phases per person-year (periods of continuous prescriptions) of preventive medications from Group 2, with a mean duration of 12.2 days per person-year, were observed in the total study population (irrespective of prescription history). When considering only those patients who received at least one prescription for the respective Group 2 preventive medication and taking into account the time after the first prescription, the mean number of phases increased to 1.15 per person-year, with a mean duration of 119.1 days per person-year (Table 4). The longest mean duration on therapy per person-year was observed for candesartan (1.48 phases with 306.3 days on therapy) and the shortest mean duration on therapy per person-year was for propranolol (1.11 phases with 70.1 days on therapy). Overall, 7.9% of the study population received the same preventive medication for more than 1 year, but most of these patients (82.9%) had discontinued the medication before the study ended. Of patients who received prescriptions for only one preventive medication (*n* = 49,909), 38.6% continued the treatment for more than 1 year.

**Table 4:** Phases of prescribed preventive medications for patients with migraine who received at least one prescription for the respective in- and off-label (Group 2) preventive medication in the German Company Sickness Fund Database 2008 to 2016

Preventive medication	Mean number of phases (per person-year)^**a**^	Mean duration of phases (days per person-year)^**b**^
All	1.15	119.1
Valproic acid	1.51	131.1
Candesartan	1.48	306.3
Bisoprolol	1.45	117.6
Onabotulinum toxin A^c^	1.41	278.5
Topiramate	1.25	138.3
Metoprolol	1.22	103.5
ARAs (‘sartans’)	1.20	274.4
Amitriptyline	1.16	92.4
Propranolol	1.11	70.1
Opipramol	1.05	81.1
Flunarizine	0.84	92.6
Lisinopril	0.67	137.5

Data shown are for prescribed Group 2 preventive medications (all preventive medications according to the German guideline for migraine [8])

^a^Calculated as the number of phases (continuous prescriptions) divided by the amount of person-time at risk (first prescription to end of enrolment)

^b^Calculated as the number of days covered by continuous prescriptions divided by the amount of person-time at risk (first prescription to end of enrolment)

^c^Prescriptions for the Allergan brand of onabotulinum toxin A only were included

*ARA* angiotensin II receptor antagonist

In 2016, the mean (SD) number of acute and emergency medication prescriptions was 2.1 (4.4) and 0.04 (0.81) per person-year, respectively, in the overall study population. The most commonly prescribed acute and emergency medications in 2016 were analgesics/NSAIDs (74.2% of acute medication prescriptions) and steroids (> 50.0% of emergency prescriptions), respectively; only 21.2% of acute prescriptions were for triptans (Table 5).

**Table 5:** Prescribed acute and emergency medications for patients with migraine in the German Company Sickness Fund Database 2016

Acute medication	Percentage of all prescriptions for acute medications
**Anti-emetics**^**a**^	**4.5%**
Metoclopramide	3.7%
**Analgesics/non-steroidal anti-inflammatory drugs**^**b**^	**74.2%**
Ibuprofen	23.7%
Metamizole	19.7%
Opioids	19.1%
Diclofenac	7.9%
Cyclooxygenase-2 inhibitors (‘coxibs’)	2.5%
Naproxen	1.1%
**Selective serotonin (5-HT1) agonists (triptans)**^**c**^	**21.2%**
Sumatriptan^d^ (all administration routes)	12.3%
Rizatriptan	4.7%
Zolmitriptan^e^ (all administration routes)	2.6%
**Emergency medication**^**f**^	**Percentage of all prescriptions for emergency medications**
Dexamethasone	51.3%
Prednisone	25.4%
Sumatriptan (subcutaneous)	13.9%
Metamizole (intravenous)	7.9%
Acetylsalicylic acid (intravenous)	1.2%

Subcutaneous sumatriptan is considered both an acute and emergency medication according to the German guideline for migraine so all identified subcutaneous sumatriptan prescriptions were included as both acute and emergency medications

^a^Also includes dimenhydrinate (0.10% of acute prescriptions) and domperidone (0.66%)

^b^Also includes acetylsalicylic acid (0.04% of acute prescriptions), paracetamol (0.10%), ergotamine (0.05%), ketoprofen (0.03%), dexketoprofen (0.20%) and other analgesics (0.92%)

^c^Also includes eleptriptan (0.18% of acute prescriptions), almotriptan (0.06%), naratriptan (0.99%) and frovatriptan (0.38%)

^d^Includes sumatriptan oral (11.8% of acute prescriptions), nasal (0.20%), rectal (0.07%) and subcutaneous (0.27%)

^e^Includes zolmitriptan oral (1.6% of acute prescriptions) and nasal (1.0%)

^f^Also includes metoclopramide (intravenous: 0.33% of emergency prescriptions)

Among patients who had been diagnosed with complicated migraine (G43.3, including chronic migraine), the mean (SD) number of acute prescriptions was 3.0 (6.8) per person-year and the mean (SD) number of emergency medication prescriptions was 0.05 (0.56) per person-year. Analgesics/NSAIDs were also more frequently prescribed than triptans in patients with a G43.3 diagnosis (57.4% vs 37.9%; the most common acute medication prescriptions included those for ibuprofen (17.4%), metamizole (14.9%) and opioids (15.4%), and the most common triptans included sumatriptan (18.0%), rizatriptan (8.6%) and zolmitriptan (7.7%). With regard to emergency medications, more than half of the prescriptions were for steroids (dexamethasone [50.9%] and prednisone [23.4%]), which was comparable to results in the overall study population (Supplementary Table 2).

Patients in the overall study population who were prescribed higher numbers of Group 1 preventive medications from different treatment classes from 2008 to 2016 also received the highest number of prescriptions for acute and emergency medications in 2016. Specifically, patients with no preventive Group 1 medication prescriptions received a mean of 1.6 (3.6) prescriptions of acute medications per person-year and a mean 0.03 (0.87) prescriptions of emergency medications per person-year. Patients with prescriptions of only one preventive medication received a mean of 3.1 (5.4) prescriptions of acute medications per person-year and a mean 0.05 (0.56) prescriptions of emergency medications per person-year. Patients with prescriptions of at least two preventive medications from different treatment classes received a mean of 5.6 (8.1) prescriptions of acute medications per person-year and a mean 0.07 (0.54) prescriptions of emergency medications per person-year.

### Patient journey

**Fig. 7:**
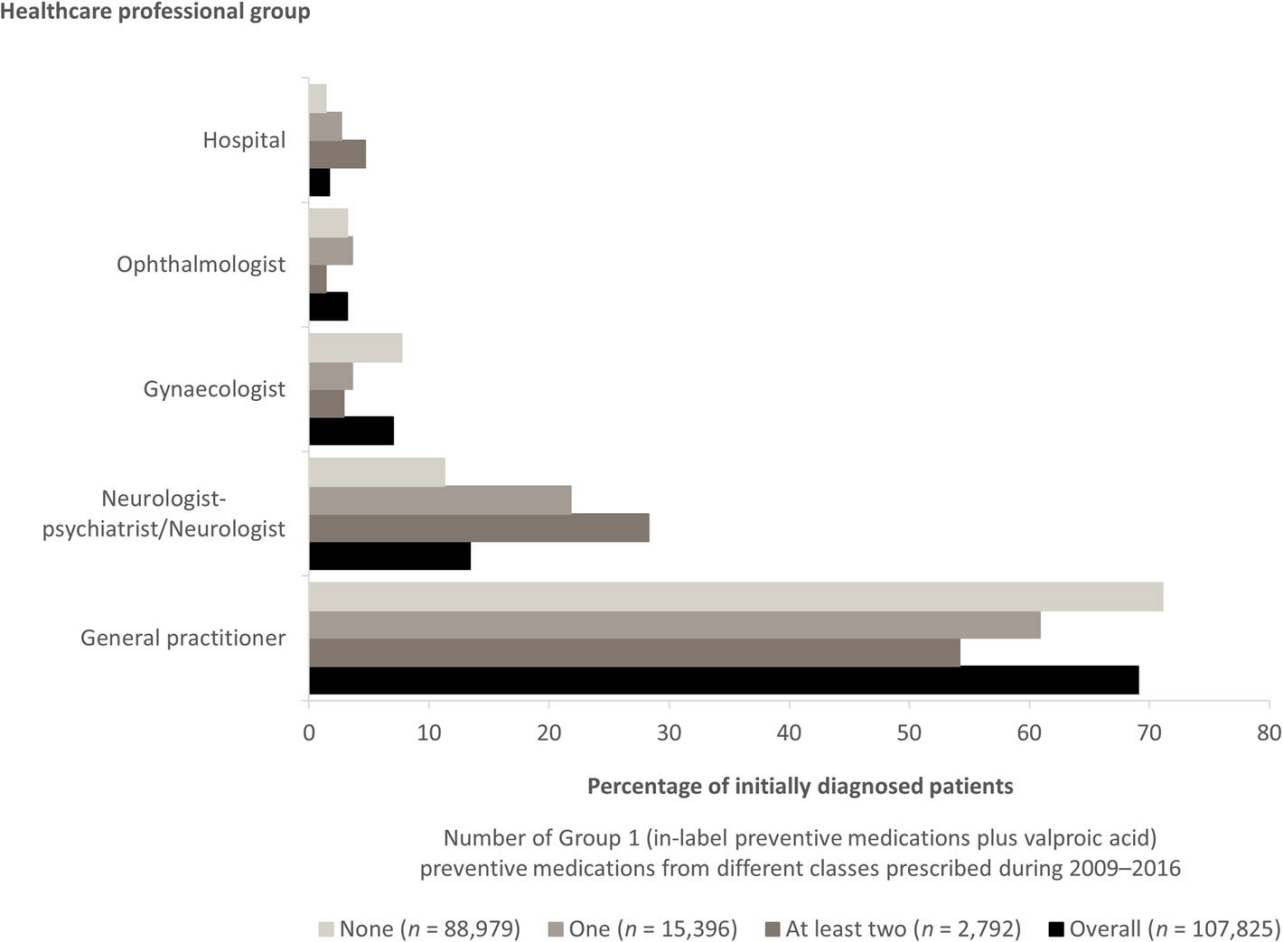
Healthcare professional group who made the first diagnosis of migraine in the German Company Sickness Fund Database 2009–2016. Data are shown for all patients (overall) and patients grouped according to the number of restricted Group 1 (propranolol, metoprolol, flunarizine, topiramate and amitriptyline, but not onabotulinum toxin A or valproic acid) preventive medications from different classes prescribed during 2009 to 2016. Data shown are for healthcare professional groups/hospital with > 1.5% of first visits overall. *n* number of patients with data

Relevant data were available from 200,658 patients with migraine in the CSFD, although numbers fluctuated by year. General practitioners most commonly diagnosed migraine in the study population from 2009 to 2016 (information based on incident cases; Fig. 7). Patients diagnosed by a neurologist were most frequently prescribed at least two preventives from 2009 to 2016; conversely, patients diagnosed by a general practitioner most frequently received no prescriptions for preventive medication during this period (Fig. 7).

Similarly, each year over the 9 years of follow-up, the most frequently visited healthcare professionals were general practitioners, followed by neurologists (including neurologist–psychiatrists) and gynaecologists (all less than one visit per person per year). Visits per person to general practitioners increased from 4.7 in the first year of follow-up to 7.9 in the ninth year of follow-up (patient numbers declined with each year of follow-up). All of the other healthcare professionals received less than one visit per person per year, with the pattern of visits to healthcare professionals remaining relatively stable over the 9-year study period. Findings with regard to the pattern of visits over the 9 years of follow-up were similar to those of the overall study population when patients with migraine were grouped according to restricted Group 1 preventive medication usage (none, only one, at least two such medications from different treatment classes). However, healthcare professional, and particularly neurologist (including neurologist-psychiatrist), visits were more frequent in patients with prescriptions for at least two preventive medications from different treatment classes than in those with no prescriptions or prescriptions for only one preventive medication (data not shown).

**Fig. 8:**
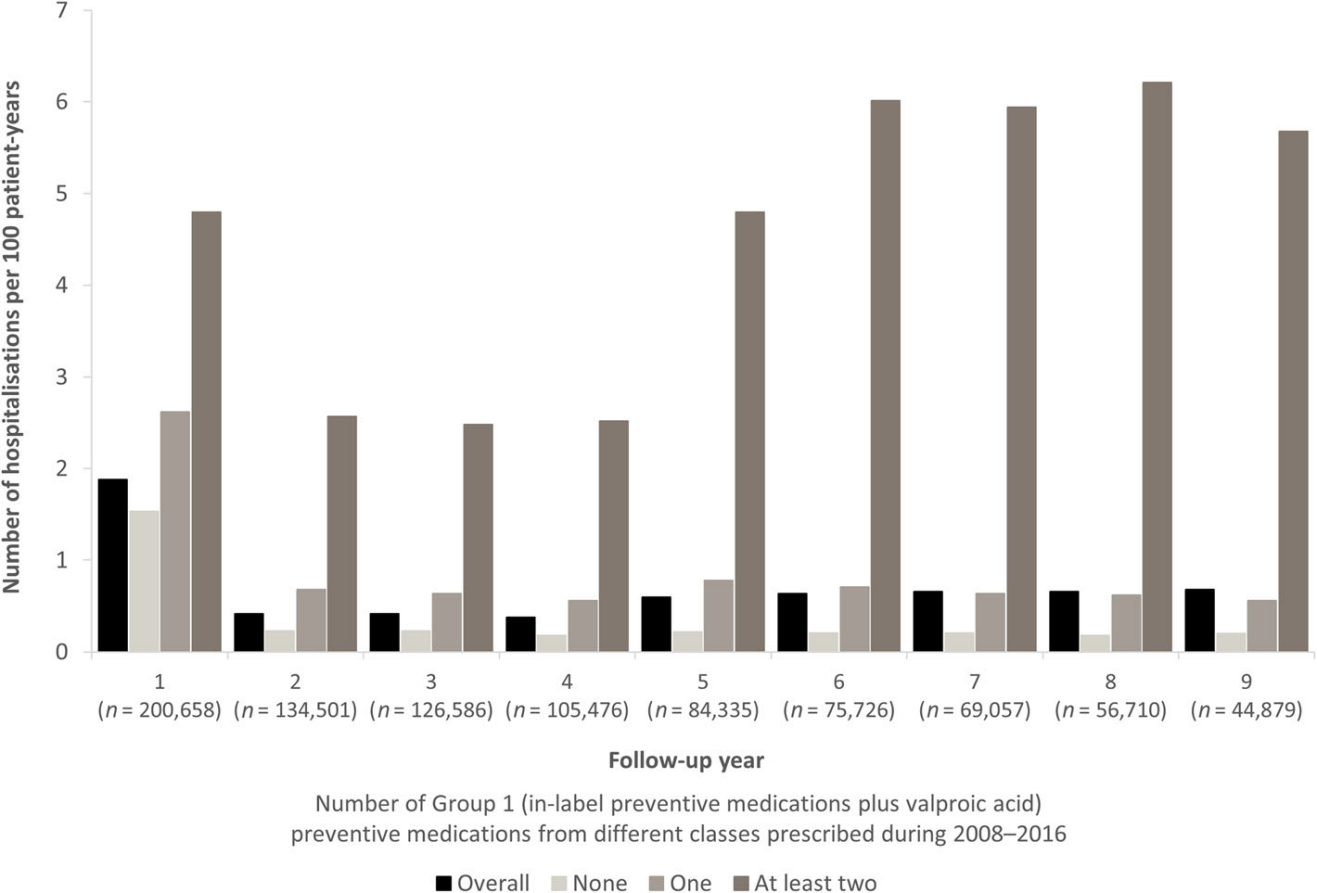
Frequency of hospitalisations of patients with migraine in the German Company Sickness Fund Database by number of in-label preventive medications from different classes. Data are shown for all patients (overall) and patients grouped according to the number of restricted Group 1 (propranolol, metoprolol, flunarizine, topiramate and amitriptyline, but not onabotulinum toxin A or valproic acid) preventive medications from different classes prescribed during 2008–2016. *n* overall number of patients each year; patients were not required to be enrolled over the total study period (2008–2016), but were required to be continuously observable. For example, a person could enter the sample later than 2008 and leave it earlier than 2016

For each year of follow-up, the number of hospitalisations per 100 person-years differed according to the number of types of restricted Group 1 preventive medications prescribed, being highest in those with a prescription for at least two different preventive medication classes (Fig. 8). In general, the number of hospitalisations per 100 person-years decreased after the first year of follow-up and then stabilised over time in most groups. The exception was in those with a prescription for at least two different preventive medication classes between 2008 and 2016 who had an increase in the number of hospitalisations per 100 person-years from year 5 of follow-up.

## Discussion

In this study, administrative claims data were analysed, revealing that the overall administrative prevalence of migraine, extrapolated to the SHI, was 4.0% in 2016. The extrapolated administrative prevalence of migraine in Germany ranged from 2.9% in 2008 to 4.1% in 2015, possibly indicating greater awareness – and hence more diagnoses – of migraine over the 9-year study period. These administrative prevalence rates are considerably lower than the migraine prevalence rates reported in German population-based analyses conducted in the early 2000s (10.6% in 2004 [12] and 13.4% in 2003–2005 [13]). The comparably lower administrative prevalence than population-based prevalence could be due to several factors. A possible explanation is that many patients with migraine experience a low frequency of attacks (≤4 headache days per month) [19] and consequently are likely to have only mild headache-associated disability, or mild impairment of health-related quality of life, work or daily activity [20]. In Germany for example, 45.2% of patients with migraine reported experiencing less than 4 headache days per month [12]. A low frequency of attacks could lead to self-medication and the patient not seeing a healthcare professional at all or after an initial diagnosis. According to an online survey of patients with migraine in Germany, the majority felt able to treat their migraine symptoms with over-the-counter medications after being diagnosed [21]. In addition, not all patients receive a migraine diagnosis. In the American Migraine Prevalence and Prevention Study, only 56.2% of participants with an ICHD 2nd edition diagnosis of migraine reported that they had ever received a medical diagnosis of the disease [22]. An analysis of data from six countries that used population-based sampling in the Eurolight survey revealed that only a minority of those with migraine consulted a healthcare professional (15.8%–33.0% across countries) [15], which might be the reason for many patients fulfilling diagnostic criteria but not receiving a medical diagnosis of migraine.

The increase in the administrative prevalence of migraine in the study period from 2008 to 2016 was observed in both men and women (from 1.0% to 1.6% in men and 4.5% to 6.1% in women). During the period 2011 to 2015, we observed little variation in the administrative incidence of migraine (between 0.19% and 0.22% in men and 0.58% and 0.64% in women). The years 2011 to 2015 were unlikely to have been affected by censorship of the data (our restriction of 2008 to 2016). Some patients diagnosed before 2008 could have been misidentified as incident cases in the first years of the study period, and some patients diagnosed late in 2016 were probably not considered as incident cases due to insufficient time to fulfil the M2Q criterion, which might explain the higher incidence rates in 2009 and 2010 and the lower rate in 2016. The opposing trend in the development of administrative prevalence and incidence over time in this study may be due to the chronic nature of migraine: while more patients with a new migraine diagnosis might enter the sample, fewer patients might leave the sample.

In the current study, a male to female ratio of close to 1:4 was observed (78.0% of the population was female). More than 40% of patients were aged 35 to 54 years. Previous population-based studies show results of a similar order of magnitude, and support the findings of the current study, despite being potentially affected by participation bias. In a 2008 review, Obermann and Katsarava [23] reported a male:female ratio of between 1:2 and 1:6 for migraine across studies; the most frequent onset of this disease occurred during the second and third decades of life, and a peak prevalence during the fourth decade of life. Lipton et al. [19] reported the highest prevalence of migraine in patients aged 30 to 39 years for both men and women, using data from the American Migraine Prevalence and Prevention study; from the published data it can be concluded that 77% of patients were women in that study. Yoon et al. [13] found most patients (both men and women) with migraine were aged between 36 and 45 years, with approximately 62% of patients being women, and the Eurolight project found a similar gender distribution in 15 European countries, with 58% of patients being women [5].

To our knowledge, this is the first study that has used claims data to evaluate changes over time in the type of healthcare professional visited by patients with migraine in Germany. In most instances, general practitioners diagnosed migraine in the index year and most frequently treated patients throughout each follow-up year. These findings are corroborated by another study that analysed patient visits during a 12-month period prior to presenting to a German specialist headache and facial pain clinic: the healthcare professionals most commonly consulted due to migraine were also general practitioners (89.5% of patients), followed by neurologists (74.9%) [24].

The most commonly prescribed acute medications in the overall study population were analgesics/NSAIDs (74.2% of acute medication prescriptions, including 23.7% ibuprofen, 19.7% metamizole and 19.1% opioids), with only 21.2% of prescriptions being for migraine-specific triptans. Patients with complicated migraine (G43.3, including chronic migraine) had a slightly higher number of acute and emergency medication prescriptions, and a higher percentage of all acute prescriptions were for triptans than was observed in the overall study population (37.9% vs 21.2%). However, analgesics/NSAIDs were still the most commonly prescribed acute medications in this patient subgroup (57.4% of all acute prescriptions). These findings suggest that migraine-specific medication is underutilised in Germany. An analysis of Eurolight data indicated that the majority of patients with migraine do not receive adequate treatment in a number of European countries, including Germany [15]. A recent report from a single specialist headache and facial pain centre in Germany showed that more than one-third of patients were not treated according to the German guideline for migraine [24]. Another study reported that approximately one-third of German patients did not receive any migraine treatment (acute or preventive) as revealed by a database analysis of 56,823 patients in 2015 [25].

Looking at preventive treatments, 22.3% of patients in the overall study population received at least one prescription for any in-label preventive medication (plus valproic acid) (Group 1 preventive medication). When both in- and off-label preventive medications (Group 2 preventive treatments) listed in the German guideline for migraine [8] were considered, the percentage was slightly higher (29.1%). Compared with the overall study population, a higher percentage of patients who had been diagnosed with complicated migraine (G43.3, including chronic migraine) received at least one prescription for in-label preventive medications plus valproic acid (38.0%; Group 1 preventive medication). Altogether, the doctors’ prescribing behaviour generally adhered to the approval and refunding status of the preventive medications in Germany.

The proportion of patients ever receiving any preventive medication in the current study is at the lower end of the range suggested as adequate by publications based on disease characteristics. The American Migraine Prevalence and Prevention study identified that 25.7% of patients met the criteria for being offered preventive medication, and an additional 13.1% should consider optionally using it [19]. More recently, the Eurolight study identified that 38.5% of patients with migraine in Germany had an attack on ≥5 days/month and would be expected to require preventive medication [15]. The potential differences between patients who might benefit from preventive medications and patients ever receiving such treatment in the current study are smaller than those reported by Ziegler et al. [24], who reported that half of the patients considered eligible for preventive medication had never received such medications.

In the current analyses, the most frequently prescribed preventive medications included beta-blockers and ARAs, which – according to the German guideline for migraine – have good and lower evidence, respectively [8]. Of those patients who received either in-label preventives (plus valproic acid) or any preventive medication (i.e. both in- and off-label preventives per German guideline for migraine), most had been prescribed only one type of drug (i.e. one drug class) during the analysis period. As few as 7.9% of patients received only one type of preventive medication lasting more than 1 year; most discontinued medications during the 9 years of follow-up, receiving a preventive medication for only a short time or only once. This finding is in general agreement with two studies conducted in the United States [26, 27]. An analysis of US administrative claims data (2005–2014) revealed that 90.3% of patients receiving first-line preventive medications were non-persistent; of these, 38.9% switched, 30.1% restarted and 31.0% discontinued treatment [26]. In another US administrative claims database analysis (2008–2015) – which included only patients with chronic migraine – oral preventive medication adherence at 12 months was higher than the rate identified in the current analyses, but still low, ranging from 17% to 20% [27]. Although an analysis of observational studies confirmed that 12-month adherence (35%–56%) and persistence (7%–55%) with preventive medications were low in patients with migraine [28], rates were usually higher than those reported in the current study. The most common reason for discontinuation of preventive medication in randomised controlled trials was reported to be adverse events [28]. Reasons for the short duration of preventive medication treatment in the current analyses are unknown.

In the current study, patients who received at least two in-label preventive medications from different classes were more likely to have reports of in- or outpatient hospitalisation. We were unable to ascertain whether hospitalisation increased the chance of receiving preventive medication or whether patients receiving multiple types of preventive medication had more severe disease that resulted in an increase in the risk of hospitalisation. Higher acute and emergency medication consumption was recorded in patients having received at least two preventive medications from different treatment classes. Healthcare resource use (migraine-related outpatient, emergency room and hospital visits, and testing) was high among patients who had discontinued at least two preventive medications because of lack of efficacy and/or tolerability in a retrospective chart review performed among neurologists, headache specialists and pain specialists in France, Germany, Italy and Spain [29]. These recent findings led the authors to conclude that there is a need for more effective prophylactic treatments to appropriately manage migraine and to reduce the associated healthcare resource use.

When deciding on a preventive treatment, a number of factors need to be considered, including existing comorbidities, as these have the potential to result in contraindications to preventive medications. As most patients in this analysis had at least one concurrent diagnosis between 2008 and 2016, it is possible that the available preventive medications were not suitable for some of the patients. However, this cannot be confirmed as the usage of preventive medication was not analysed according to concurrent diagnoses. Nevertheless, some patients may have benefitted from alternative therapeutic options. Since the end of the analysis period (2016), monoclonal antibodies targeting CGRP or its receptor have expanded the preventive treatment options for patients with migraine in Germany. Further studies are needed to determine the impact of this expanded portfolio.

### Limitations

This analysis shares the limitations of all database analyses, particularly those involving the SHI (see [16] for details). Limitations more specific to this retrospective database study, which analysed data between 2008 and 2016, are discussed here. Incident cases were approximated since they were defined by at least 1 year without a diagnosis of migraine before the index diagnosis. It is therefore possible that some patients had been diagnosed prior to 2008, but had not received any migraine-associated medical care in the year before the index diagnosis. Additionally, patients who did not consult a healthcare professional regarding their migraine were not included in the analyses. The statistics for outpatient care in hospitals must be regarded as underestimated since relevant codes could be found in only one-third of the cases (data not shown); acute medication usage must be viewed as heavily underestimated since this medication group contains common over-the-counter medications (e.g. acetylsalicylic acid, ibuprofen and paracetamol).

Also, it was not possible to ensure that medications classified as acute or preventive against migraine were, indeed, for the management of migraine, as most are approved for several indications. To mitigate this limitation, a diagnosis of migraine needed to be documented in the in- or outpatient, or sick-leave data within the same quarter as the medication prescription. However, due to the high rates of concomitant diseases, the percentages of non-specific migraine medications, such as opioids, need to be interpreted within this context.

With increasing number of lines of therapies, the number of patients with data became smaller, which should be taken into account when interpreting the data.

The trend of an increase in prevalence each year was not seen between 2015 and 2016, which was most likely due to bias introduced by our M2Q criterion; this meant that cases incident later in 2016 may not have been recognised by this criterion. This effect was more prominent when the incidence of migraine was considered, and incidence rates in 2016 were biased to be low due to the M2Q criterion. Likewise, incidence rates from 2009 have to be considered with care. Although 1 year of enrolment without a migraine diagnosis was required for our estimation of incidence, incident cases in the years following 2009 may have had a longer period after enrolment without a diagnosis of migraine than cases in the first possible year of enrolment. This may explain the higher incidence rates in 2009, and possibly also in 2010.

## Conclusion

The administrative prevalence of migraine in these analyses was lower than historically reported population-based prevalence rates, suggesting that many patients with migraine do not seek medical care. Preventive medication was received by less than one-third of patients, with most prescribed only one such medication and few continuing preventive treatment beyond 1 year. Patients who did have prescriptions for at least two different preventive therapy classes during the study period were the most likely to use other healthcare resources (healthcare professional visits and acute and emergency medications). These outcomes suggest that there is scope for improvement in migraine management in Germany.

## Supplementary information

**Additional file 1: Figure S1.** Age distribution by gender in the German Company Sickness Fund database (CSFD) in 2016 and the entire insured population of the German Statutory Health Insurance (SHI) system in 2016

**Additional file 2: Table S1.** Summary of acute, emergency and prophylactic medications in the German Company Sickness Fund Database^a^

**Additional file 3: Table S2.** Prescribed acute medications for patients who had been diagnosed with complicated migraine (G43.3, including chronic migraine) in the German Company Sickness Fund Database 2016 (*N* = 2458)

## Data Availability

The data analysed are subject to national data protection laws and are only available upon formal request. The responsible contact person is Dr Heiko Friedel, Leader of the Health Outcome Research and Health Economics Department, Team Gesundheit GmbH, 45,128 Essen, Germany. All data were anonymised and prepared in accordance with a confirmed data privacy concept. The cooperating sickness funds agreed upon temporary preparation and storage of their data for this purpose. Access to these data by other parties is not permitted.

## Abbreviations

ACEi: Angiotensin-converting enzyme inhibitor
ARA: Angiotensin II receptor antagonist
ATC: Anatomical Therapeutic Chemical
CGRP: Calcitonin gene-related peptide
CI: Confidence interval
CSFD: German Company Sickness Fund database
ICD-10-GM: International Classification of Diseases, 10th revision, German modification
ICHD: International Classification of Headache Disorders
PZN: *Pharmazentralnummer*
SD: Standard deviation
SHI: Statutory Health Insurance
