# Supplementary figures and images for: Administrative prevalence and incidence, characteristics and prescription patterns of patients with migraine in Germany: a retrospective claims data analysis
Source: J Headache Pain. 2020 Jul 6;21(1):85. doi: 10.1186/s10194-020-01154-x (PMC7339552; doi:10.1186/s10194-020-01154-x)

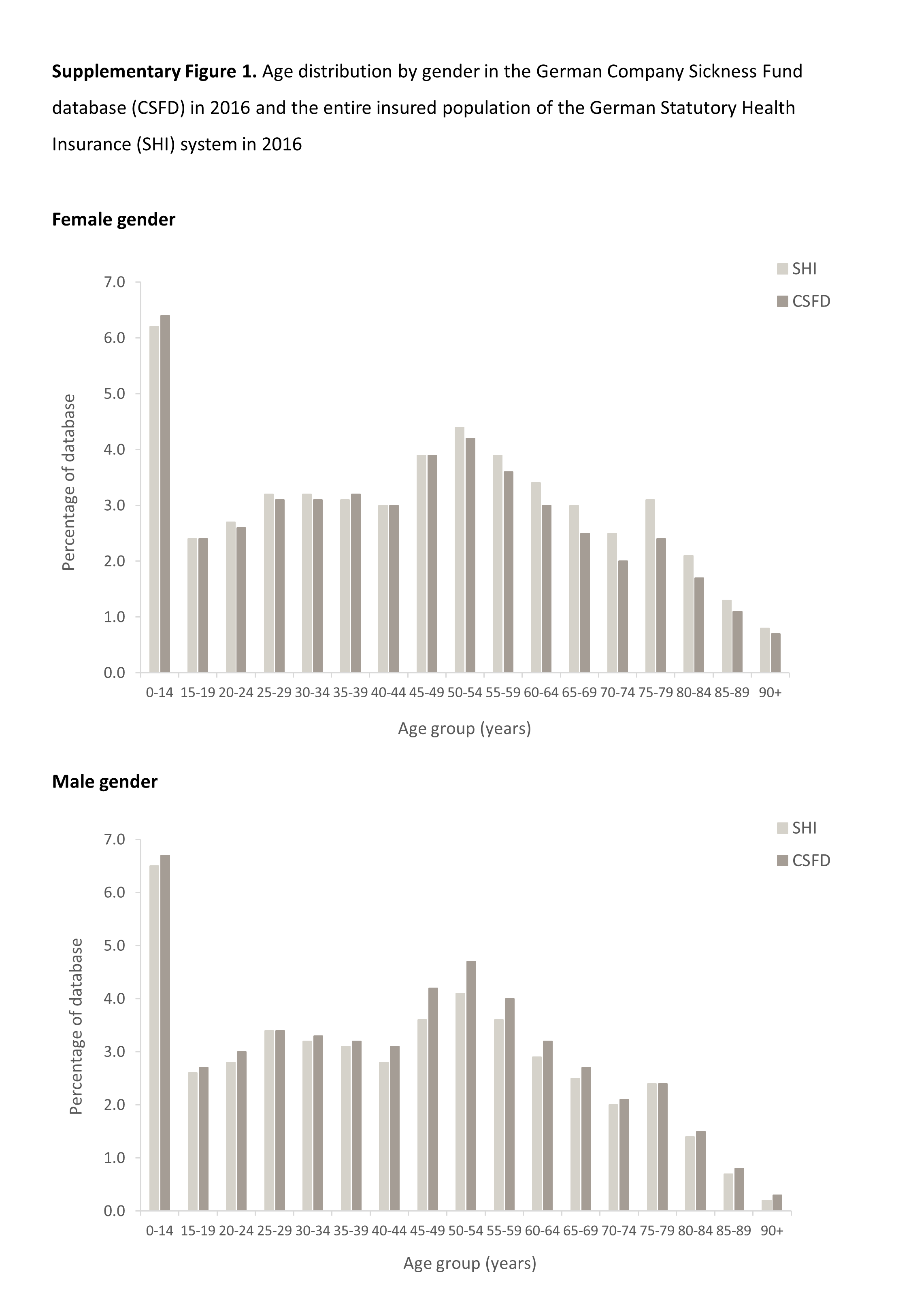

Supplement: Supplementary file 1 — Additional file 1: Figure S1. Age distribution by gender in the German Company Sickness Fund database (CSFD) in 2016 and the entire insured population of the German Statutory Health Insurance (SHI) system in 2016 [file 10194_2020_1154_MOESM1_ESM.tif]
