# Supplementary material for: Administrative prevalence and incidence, characteristics and prescription patterns of patients with migraine in Germany: a retrospective claims data analysis
Source: J Headache Pain. 2020 Jul 6;21(1):85. doi: 10.1186/s10194-020-01154-x (PMC7339552; doi:10.1186/s10194-020-01154-x)
Supplement: Supplementary file 2 — Additional file 2: Table S1. Summary of acute, emergency and prophylactic medications in the German Company Sickness Fund Databasea [file 10194_2020_1154_MOESM2_ESM.docx]

**Supplementary Table 1** Summary of acute, emergency and prophylactic medications in the German Company Sickness Fund Database^a^

| **Medication** | |
| --- | --- |
| **Acute medication** |  |
| Metoclopramide | Naproxen |
| Dimenhydrinate | Sumatriptan |
| Domperidone | Oral |
| Opioids | Nasal |
| Acetylsalicylic acid | Rectal |
| Ibuprofen | Subcutaneous |
| Metamizole | Eleptriptan |
| Diclofenac | Rizatriptan |
| Paracetamol | Zolmitriptan |
| Acetylsalicylic acid (combinations) | Oral |
| Ergotamine | Nasal |
| Cyclo-oxygenase-2 inhibitors (‘coxibs’) | Almotriptan |
| Ketoprofen | Naratriptan |
| Dexketoprofen | Frovatriptan |
| Other analgesics |  |
| **Emergency medication** |  |
| Metoclopramide (intravenous) | Sumatriptan (subcutaneous) |
| Metamizole (intravenous) | Prednisone |
| Acetylsalicylic acid (intravenous) | Dexamethasone |
| **Preventive medication** |  |
| Propranolol | Amitriptyline |
| Metoprolol | Onabotulinum toxin A |
| Bisoprolol | Opipramol |
| Flunarizine | Magnesium compounds |
| Valproic acid | Lisinopril |
| Topiramate | Angiotensin II receptor antagonists (‘sartans’) |

^a^All listed migraine medications were selected based on the guideline of the German Migraine and Headache Society and the German Society of Neurology [8]) and are included in the database analyses. Subcutaneous sumatriptan is considered both an acute and emergency medication according to this guideline and so all identified subcutaneous sumatriptan prescriptions were included as both acute and emergency medications
