# Supplementary material for: Administrative prevalence and incidence, characteristics and prescription patterns of patients with migraine in Germany: a retrospective claims data analysis
Source: J Headache Pain. 2020 Jul 6;21(1):85. doi: 10.1186/s10194-020-01154-x (PMC7339552; doi:10.1186/s10194-020-01154-x)
Supplement: Supplementary file 3 — Additional file 3: Table S2. Prescribed acute medications for patients who had been diagnosed with complicated migraine (G43.3, including chronic migraine) in the German Company Sickness Fund Database 2016 (N = 2458) [file 10194_2020_1154_MOESM3_ESM.docx]

**Supplementary Table 2**  Prescribed acute medications for patients who had been diagnosed with complicated migraine (G43.3; including chronic migraine) in the German Company Sickness Fund Database 2016 (N=2458)

| **Acute medication** | **Percentage of all prescriptions for acute medications** |
| --- | --- |
| **Anti-emetics^a^** | **4.7%** |
| Metoclopramide | 3.9% |
| **Analgesics/non-steroidal anti-inflammatory drugs^b^** | **57.4%** |
| Ibuprofen | 17.4% |
| Metamizole | 14.9% |
| Opioids | 15.4% |
| Diclofenac | 5.5% |
| Cyclooxygenase-2 inhibitors (‘coxibs’) | 2.1% |
| Naproxen | 1.7% |
| **Selective serotonin (5-HT1) agonists (triptans)^c^** | **37.9%** |
| Sumatriptan^d^ (all administration routes) | 18.0% |
| Rizatriptan | 8.6% |
| Zolmitriptan^e^ (all administration routes) | 7.7% |
| Naratriptan | 1.7% |
| Frovatriptan | 1.0% |
| **Emergency medication** | **Percentage of all prescriptions for emergency medications** |
| Dexamethasone | 50.9% |
| Prednisone | 23.4% |
| Sumatriptan (subcutaneous) | 15.5% |
| Metamizole (intravenous) | 5.6% |
| Metoclopramide (intravenous) | 1.8% |
| Acetylsalicylic acid (intravenous) | 2.7% |

^a^Also includes dimenhydrinate (0.15% of acute prescriptions) and domperidone (0.61%)

^b^Also includes acetylsalicylic acid (0.04% of acute prescriptions), paracetamol (0.07%), ergotamine (0.13%), dexketoprofen (0.14%) and other analgesics (0.59%)

^c^Also includes eleptriptan (0.72% of acute prescriptions) and almotriptan (0.14%)

^d^Includes sumatriptan oral (16.7% of acute prescriptions), nasal (0.45%), rectal (0.07%) and subcutaneous (0.81%)

^e^ Includes zolmitriptan oral (4.8% of acute prescriptions) and nasal (2.9%)

Subcutaneous sumatriptan is considered both an acute and emergency medication according to the German guideline for migraine and so all identified subcutaneous sumatriptan prescriptions were included as both acute and emergency medications
